# BAT-tling oxidative stress through BCAA catabolism

**DOI:** 10.1093/lifemeta/loae028

**Published:** 2024-06-28

**Authors:** Maria Delgado-Martin, Qiaoqiao Zhang, Lawrence Kazak

**Affiliations:** Rosalind & Morris Goodman Cancer Institute and Department of Biochemistry, McGill University, Montreal, QC, H3A 1A3, Canada; Department of Biochemistry, McGill University, Montreal, QC, H3G 1Y6, Canada; Rosalind & Morris Goodman Cancer Institute and Department of Biochemistry, McGill University, Montreal, QC, H3A 1A3, Canada; Department of Biochemistry, McGill University, Montreal, QC, H3G 1Y6, Canada; Rosalind & Morris Goodman Cancer Institute and Department of Biochemistry, McGill University, Montreal, QC, H3A 1A3, Canada; Department of Biochemistry, McGill University, Montreal, QC, H3G 1Y6, Canada

**Elevated circulating levels of branched-chain amino acids (BCAAs) are associated with the development of type 2 diabetes and obesity, diseases that can be countered by the energy dissipating (thermogenic) function of brown adipose tissue (BAT). In a recent study published in *Cell*, Verkerke and colleagues report that BAT promotes insulin sensitivity in the liver by coupling antioxidant homeostasis with BCAA catabolism, an effect that is independent of its thermogenic properties.**

The molecular features of brown and beige adipocytes endow these cells with a unique capacity to dissipate chemical energy as heat (thermogenesis). Moreover, the presence of brown adipose tissue (BAT) in adult humans and its association with metabolic health [1] have driven major attempts at trying to understand the molecular mechanisms driving adipocyte thermogenesis in order to target these pathways to offset obesity-accelerated diseases. Evidence from preclinical models indicates that thermogenic fat disproportionately affects glucose homeostasis over body weight [2]. Likewise, initial prospective clinical studies have shown that increasing the metabolic activity of thermogenic fat is associated with improved insulin sensitivity and cardiometabolic health, even without weight loss [3]. One major unresolved question in the field is whether the metabolic benefits of brown and beige adipocytes result from their thermogenic property or are independent of it.

Branched-chain amino acids (BCAAs: valine, leucine, and isoleucine) account for ~20% of protein intake and their impaired oxidation in peripheral organs leads to high circulating BCAA levels that are markers of obesity, insulin resistance, and type 2 diabetes [4]. Contrary to other amino acids, BCAAs are poorly metabolized in the liver due to the low hepatic expression of mitochondrial branched-chain aminotransferase (BCAT2), the first enzyme in the BCAA catabolic pathway [5]. BCAA transamination through BCAT2 generates branched-chain α-keto acids (BCKAs) that undergo oxidative decarboxylation by the branched-chain α-keto acid dehydrogenase complex (BCKDH) into distinct acyl-CoA derivates depending on the individual BCAA. For example, valine is converted into succinyl-CoA, entering the citric acid cycle and supporting gluconeogenesis. Leucine, being ketogenic, is transformed into acetyl-CoA and acetoacetate, providing energy during fasting. Isoleucine contributes to both glucogenic and ketogenic pathways, producing acetyl-CoA and succinyl-CoA for energy and glucose synthesis.

Since BCAAs cannot be processed in the liver, their catabolism falls on other organs, such as adipose tissue and skeletal muscle. Interestingly, during thermogenesis, glucose contributes the vast majority (approximately 70%) of the total carbon influx into BAT under cold conditions [6]. Therefore, since BCAAs are not a major carbon source for BAT, they do not appear to be a major fuel for thermogenesis.

**Figure 1:**
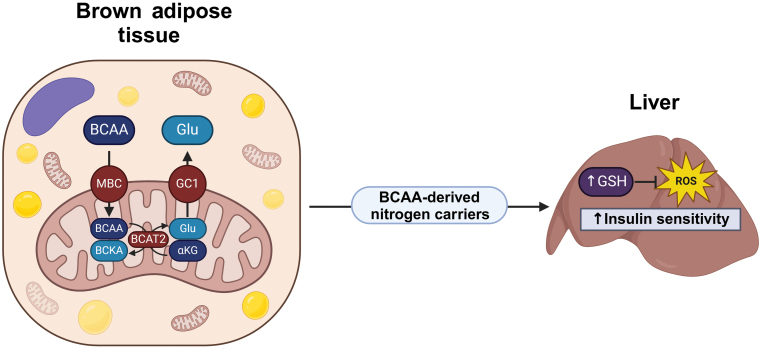
Schematic diagram of branched-chain amino acid (BCAA) catabolism in BAT and its relationship with liver function and oxidative stress. BCAAs in brown adipocytes are transported into the mitochondrial matrix through the mitochondrial BCAA carrier (MBC), where they are transaminated with α-ketoglutarate (αKG) by the mitochondrial branched-chain aminotransferase (BCAT2) to generate branched-chain α-keto acids (BCKAs) and glutamate (Glu). These and other BCAA-derived nitrogenated metabolites, like glutathione (GSH), go into the circulation and reach the liver to blunt oxidative stress and improve insulin sensitivity. Created with Biorender.com.

BAT plays a key role in the clearance of BCAAs from the circulation and requires the mitochondrial BCAA carrier (MBC, previously known as SLC25A44) [7, 8]. Previously, the Kajimura group reported that BCAA catabolism is required for BAT thermogenesis and systemic BCAA clearance in mice and humans [8]. In their most recent work, Verkerke *et al*. [9] employed a BAT-specific *MBC* knockout mouse model to provide evidence that MBC is required to support BCAA catabolism and maintain insulin signaling in the liver by offsetting oxidative stress independent of any effects on energy expenditure and body weight (Fig. 1).

Verkerke *et al*. performed liquid chromatography-mass spectrometry (LC-MS) metabolomics from extracellular fluids isolated from interscapular BAT and epididymal white adipose tissue (WAT). Metabolomics analysis showed an enrichment of BCKAs, glutamate (Glu), N-acetylglutamate, N-acetylasparatate, and glutathione in BAT-derived extracellular fluids, metabolites that participate in BCAA catabolism. Next, the authors found that BCAAs are the principal nitrogen source for the synthesis of Glu and alanine (Ala) because ^15^N-BCAA tracing experiments resulted in 60% of these amino acid pools being ^15^N-labeled. On the other hand, under the conditions where 90% of BCKAs were ^13^C-labeled, citric acid cycle intermediates were at most 10% labeled.

The Kajimura group previously demonstrated that MBC transports BCAAs into the mitochondrial matrix, where they are deaminated by BCAT2, producing Glu and Ala [8]. Using ^15^N-BCAA tracing experiments in the context of *MBC* knockout (*MBC* KO) and genetic and pharmacological inhibition of BCAT2 in brown adipocytes, Verkerke *et al*. showed a significant reduction in the levels of ^15^N-BCAA-derived metabolites with both inhibition approaches. These results substantiate the key role of MBC and BCAT2 in BCAA catabolism within the same pathway in brown adipocytes.

Next, the authors performed MBC interactome analysis, identifying 284 mitochondrial proteins associated with MBC that, according to pathway analysis, were significantly involved in BCAA catabolism. This gives rise to the idea of a BCAA metabolon for efficient BCAA metabolism. Consistent with this hypothesis, when BCAT2 was the bait, 95% of the BCAT2-associated proteins were shared with the MBC-associated proteome. The Glu carrier (GC1, encoded by *Slc25a22*) was identified as being proximal to MBC, indicating that GC1 could support BCAA catabolism by effluxing Glu derived from BCAA transamination. Consistent with this hypothesis, the authors showed that single deletion of GC1 or MBC individually blunted the thermogenesis of brown adipocytes in response to BCAA supplementation and acute norepinephrine treatment, while combined deletion showed an even stronger effect. Moreover, the functional interaction between MBC and GC1 was further confirmed by a reduction in ^15^N-BCAA derived metabolites when either carrier was absent, again with an exacerbated effect with combined deletion.

Silencing *MBC* expression in all tissues (mice lacking *MBC*, that is, *MBC*-KD) doubled fasting serum BCAA levels compared to control mice (from ~0.2 mmol/L in control mice to ~0.4 mmol/L in *MBC*-KD mice) and nearly doubled serum BCAA concentrations 2 h after an oral challenge (from ~0.6 mmol/L in control mice to ~1 mmol/L in *MBC*-KD mice). ^15^N-labeled BCAA tracing studies showed reduced synthesis of BCAA-derived metabolites in *MBC*-KD mice compared to control mice. Importantly, *MBC*-KD mice exhibited glucose and insulin intolerance independent of body weight. Mice genetically lacking *MBC* in Ucp1^+^ adipocytes (*MBC^UCP1^* KO) displayed a reduced capacity to clear BCAAs from the circulation and showed impaired insulin tolerance independent of major changes in whole-body energy expenditure or body weight. The authors found that in response to insulin administration, the liver, but not adipose tissue or skeletal muscle, displayed reduced AKT^Ser473^ phosphorylation, reduced phosphorylation of AKT substrates, and reduced activity of pyruvate dehydrogenase. Thus, BCAA metabolism in Ucp1^+^ cells somehow controls liver insulin signaling.

Metabolomics identified a reduction in BCAA-derived metabolites (glutamic acid and glutathione) in serum and an increase in liver oxidative stress markers in *MBC^UCP1^* KO mice compared to control mice. Glutathione supplementation for 10 days restored insulin tolerance in MBC^UCP1^ KO mice and liver insulin signaling to the levels seen in control mice, highlighting the critical role of glutathione in mitigating oxidative stress and maintaining insulin sensitivity. These findings indicate that impaired BCAA flux and catabolism in Ucp1^+^ cells leads to increased oxidative stress and insulin resistance, particularly affecting liver function.

The authors next explored the physiological contexts where BCAA metabolism might be altered in BAT. They found reduced BCAA oxidation in BAT of high-fat diet-fed mice. Furthermore, ^15^N-BCAA tracing experiments demonstrated significantly lower levels of BCAA-derived metabolites in obese mice compared to lean controls. These findings suggest that reduced BCAA catabolism in BAT during obesity leads to decreased synthesis of metabolites crucial for maintaining redox balance and metabolic function. Cold acclimation enhanced BCAA uptake and metabolite synthesis in BAT, highlighting the tissue’s dynamic response to temperature changes. In humans, cold exposure increased circulating glutathione levels in individuals with high BAT activity (as assessed by positron emission tomography-computed tomography with ^18^F-fluorodeoxyglucose (^18^FDG-PET)), suggesting a link between BAT activation and glutathione synthesis.

These results give rise to new questions regarding the involvement of BAT in BCAA metabolism and its connection to insulin signaling in the liver. First, Verkerke *et al*. describe a way of communication between BAT and the liver through BCAA-derived metabolites. The authors convincingly demonstrate the essential role of Ucp1^+^ cells in supplying BCAA-derived metabolites that the liver requires to support whole-body insulin sensitivity. However, skeletal muscle is a major site of BCAA metabolism. So, what role do BCAA-derived metabolites arising from skeletal muscle play in physiology? Second, Verkerke *et al*. showed that following norepinephrine treatment, both valine and α-ketoisovaleric acid (KIV) supplementation increased respiration of wild-type brown adipocytes. These data indicate that there is a mechanism that links adrenergic signaling to BCAA catabolism and potentiation of thermogenesis. Future exploration into the stage of BCAA metabolism where norepinephrine imparts its signal would provide additional mechanistic insight into the BCAA catabolic pathway. Third, the authors convincingly show that BCAA catabolism in BAT controls whole-body insulin signaling independent of whole-body energy expenditure. However, discerning the effect of BAT thermogenesis on whole-body energy expenditure is challenging. A prime example comes from mice with germline *Ucp1* deletion, a model with powerful BAT disruption, which does not exhibit reduced cold-induced whole-body energy expenditure [10], possibly due to compensation from skeletal muscle shivering. Indeed, the authors demonstrated that BCAA supplementation potentiates thermogenesis in a cell-autonomous manner [8, 9]. Thus, the thermogenic and non-thermogenic effects of BCAAs on BAT function may not be mutually exclusive, and whether these effects are fully separable remains to be determined. Fourth, AAV-mediated *MBC* silencing in BAT has been shown to decrease noradrenaline-induced heat production and blunt body temperature maintenance in the cold [8]. In Verkerke *et al*. [9], these thermogenic effects were not found using *Ucp1*-driven Cre recombinase to delete *MBC*. The cause of these differences is currently unknown, but could be a result of the distinct genetic approaches used to disrupt *MBC*. Lastly, *Bckdha* deletion in Ucp1^+^ cells (*Bckdha^UCP1^* KO) decreased noradrenaline-stimulated heat production in BAT, impaired body temperature maintenance in the cold, and increased body weight compared to control mice [8]. Since BCKDHA acts downstream of MBC, the thermogenic impairment of *Bckdha*^*UCP1*^ KO, but not *MBC^UCP1^* KO, mice suggest that BCKDHA has MBC-independent functions.

In sum, the study of Verkerke *et al*. provides evidence for metabolic benefits from a BAT-liver axis. Defective BCAA mitochondrial metabolism in BAT impairs insulin signaling in the liver. MBC is required in BAT for BCAA-derived synthesis of non-essential amino acids and their derived metabolites, including glutathione. Moreover, the authors show that glutathione supplementation reverses glucose intolerance of mice genetically lacking *MBC* in BAT, strongly indicating that BAT mediates antioxidant homeostasis to support metabolic health. Collectively, the intriguing results of Verkerke *et al*. bring new research opportunities to the BAT field, highlighting the importance of its function as a secretory organ that contributes to whole-body metabolic homeostasis.
